# Limits to the rate of information transmission through the MAPK pathway

**DOI:** 10.1098/rsif.2018.0792

**Published:** 2019-03-06

**Authors:** Frederic Grabowski, Paweł Czyż, Marek Kochańczyk, Tomasz Lipniacki

**Affiliations:** 1 Faculty of Mathematics, Informatics and Mechanics, University of Warsaw, Warsaw, Poland; 2 Mathematical, Physical and Life Sciences Division, University of Oxford, Oxford, UK; 3 Institute of Fundamental Technological Research, Polish Academy of Sciences, Warsaw, Poland

**Keywords:** cellular signal transduction, pulsatile stimulation, pulse–interval transcoding, bandwidth, representation problem

## Abstract

Two important signalling pathways of NF-κB and ERK transmit merely 1 bit of information about the level of extracellular stimulation. It is thus unclear how such systems can coordinate complex cell responses to external cues. We analyse information transmission in the MAPK/ERK pathway that converts both constant and pulsatile EGF stimulation into pulses of ERK activity. Based on an experimentally verified computational model, we demonstrate that, when input consists of sequences of EGF pulses, transmitted information increases nearly linearly with time. Thus, pulse-interval transcoding allows more information to be relayed than the amplitude–amplitude transcoding considered previously for the ERK and NF-κB pathways. Moreover, the information channel capacity C, or simply bitrate, is not limited by the bandwidth *B* = 1/*τ*, where *τ* ≈ 1 h is the relaxation time. Specifically, when the input is provided in the form of sequences of short binary EGF pulses separated by intervals that are multiples of *τ*/*n* (but not shorter than *τ*), then for *n* = 2, *C* ≈ 1.39 bit h^−1^; and for *n* = 4, *C* ≈ 1.86 bit h^−1^. The capability to respond to random sequences of EGF pulses enables cells to propagate spontaneous ERK activity waves across tissue.

## 1. Introduction

Cells secrete and recognize chemical signals in order to coordinate their actions at a supercellular level. In addition to chemical identity, quantifiable features of the signal such as amplitude or duration are usually relevant for eliciting a proportionate physiological cell response. The amplitude of an input signal can be translated into the amplitude or duration of activity of an effector protein such as a transcription factor. It has been shown for the nuclear factor kappa–light chain enhancer of activated B cells (NF-κB) system that the response in a population of cells is proportional to the product of signal amplitude and its duration [1]. Some signalling pathways that exhibit oscillatory behaviour transcode the input amplitude to the frequency of effector pulses [2]. Such an ability has been recently demonstrated in the mitogen-activated protein kinase (MAPK) pathway, where, in response to extracellular epidermal growth factor (EGF) (input) of constant amplitude, extracellular signal-regulated kinase (ERK) (effector) exhibits activity pulses with constant amplitude but frequency and pulse duration determined by concentrations of EGF [3]. In the MAPK system, the amplitude-to-frequency transcoding is enabled by a particular topology of feedback loops and associated time scales [4].

Molecular interpretation of pulsatile (or more complex) temporal codes of effector molecules is an area of active research [5–7]; however, less attention has been devoted to the characterization of responses of signalling systems challenged with time-varying inputs. It has been demonstrated that both the NF-κB and the MAPK/ERK pathways respond in a pulsating manner to periodic pulses of cytokines, tumour necrosis factor (TNF) and EGF, respectively [8,9]. Experimental and computational analysis of responses to pulsating inputs has shown that the ability of the NF-κB system to respond to a series of TNF pulses is inherently limited by the relaxation time *τ* associated with oscillation-generating negative feedback [10,11]. Information transmission through both the NF-κB and the MAPK/ERK pathways has been analysed within the generic framework of information theory [12,13], revealing that these pathways are able to transmit about 1 bit of information about a constant stimulus concentration [14–17].

**Figure 1.**
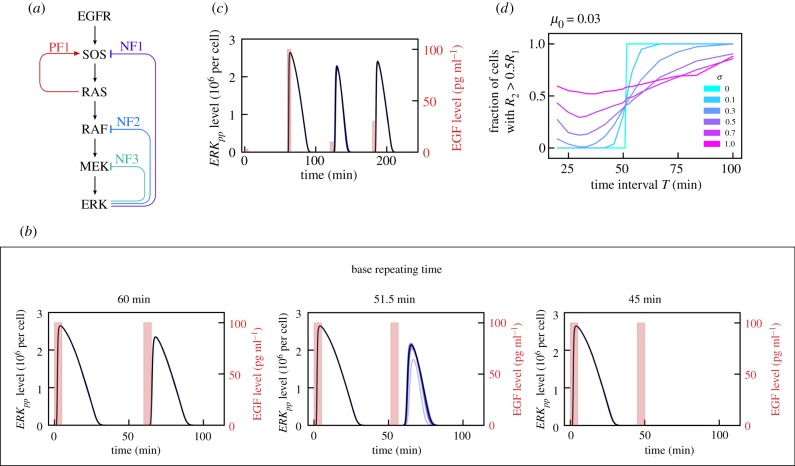
The MAPK pathway and analysis of ERK responses to repeated EGF pulses. (*a*) Schematic diagram of the MAPK pathway. As analysed in [4], PF1 and NF1 lead to relaxation oscillations, whereas NF2 and NF3 shape the time profile of the pulse of active ERK (*ERK_pp_*). (*b*) *ERK_pp_*(*t*) profile in response to two 5 min long 100 pg ml^−1^ EGF pulses separated by *T* = 45, 51.5, 60 min. Black bold line: deterministic model trajectory; thin blue lines: 10 stochastic trajectories (visible only for *T* = 51.5 min). (*c*) *ERK_pp_*(*t*) profile in response to four EGF pulses repeated every 60 min with successive amplitudes 3, 100, 10, 30 pg ml^−1^. (*d*) Fraction of simulated cells for which *R*_2_ > 0.5*R*_1_, as a function of the time interval *T* between EGF pulses. The fraction is calculated based on at least 500 independent numerical simulations (number of simulations for *T* close to *τ* was up to 5000) performed assuming lognormal distributions logN(*μ_i_*, *σ*^2^) of protein levels (and first-order reaction coefficients) with mean *μ_i_* equal to the default value of a given variable, and six different values of *σ*. EGFR, epidermal growth factor receptor; MEK, mitogen-activated protein kinase kinase; NF, negative feedback; PF, positive feedback; SOS, son of sevenless. (Online version in colour.)

In this work, we present a computational analysis of information relay within an experimentally verified model of the MAPK/ERK pathway [4], calibrated on MCF10A cells [3], where a fast positive feedback loop (involving RAS and SOS) is nested within a slow negative feedback loop (involving ERK and SOS; see figure 1*a*). Such network topology allows both to transcode constant EGF stimulation to periodic ERK activity pulses and to transcode analogous EGF pulses to nearly digital pulses of ERK activity. Based on the model, we approach the following representation problem: What is the signal transcoding strategy that allows the maximum amount of information to be transmitted through a given transmission channel in a given time span or to achieve the highest information transmission rate in the long term? We propose to code input information in the form of sequences of short digital EGF pulses. Then, we estimate the maximum transmission rate with respect to the noise strength. In particular, we demonstrate that when input information is encoded in intervals between subsequent EGF pulses, then the information channel capacity, computed as the maximum mutual information (MI) rate, may exceed channel bandwidth, *B* = 1/*τ*. By theoretical and numerical analysis of transmission of binary EGF sequences with varying inter-pulse intervals we demonstrate that the MAPK/ERK pathway is able to transmit more than 1 bit per hour.

## 2. Methods

### 2.1. Parameters and simulations

To perform our analyses, we employed the computational model described in [4], which has been amended with extrinsic noise and perturbed according to stimulation protocols, characterized by the following parameters:

— pulse duration: 5 min, pulse amplitude: 0 or 100 pg ml^−1^ (unless otherwise specified);
— (square of) the second parameter of the lognormal distribution describing additive noise: *σ*_0_ = 1.0;
— (square of) the second parameter of the lognormal distribution describing cell-specific noise: *σ* = 0, 0.1, 0.3, 0.5, 0.7, 1.0;
— basic term to determine the first parameter of the lognormal distribution describing cell-specific noise: *μ*_0_ = {0, 0.01, 0.03, 0.1, 0.3};
— number of pulses: 6 or 8, inter-pulse interval: *T* = 15, 20, 30, 40, 50, 60, 70, 80 min;
— number of simulations for a given input sequence of pulses: *M* = 1000.

All parameters of the MAPK model are provided in electronic supplementary material, table S1. Abundances of molecules of each pathway component were drawn from lognormal distributions logN(*μ_i_*, *σ*) with median *μ_i_* equal to a default value for this component. Also, all pseudo-first-order reaction parameters, which are proportional to the level of an implicit enzyme, were independently drawn from a lognormal distribution. These randomly set parameters of the MAPK pathway are written in green in electronic supplementary material, table S1. For each considered combination of inter-pulse interval *T*, noise strength *σ* and specific input sequence of pulses, we generated *M* cell-specific random sets of parameters (as described above) and simulated model dynamics of each cell deterministically using an adaptive ODE solver embedded in BioNetGen [18]. To simulate the effect of the additive noise of different strengths, we post-processed the obtained *M* trajectories of *ERKpp*(*t*).

### 2.2. Mutual information calculation and maximization

Estimation of the MI defined in equation (3.2) has been performed as proposed by Kraskov *et al*. [19]. In this approach (see [19], eqn (8)), effectively the output probability distributions, *Y*_i_, and marginal distribution, *Y*, are estimated based on the distance to the *k*th nearest neighbour and on the number of points within this distance. For all estimates described in this article, we have set the number of nearest neighbours *k* = 15, and assumed that distances in input space *X* are much larger than those in *Y* space (which implies that in eqn (8) from [19] we set *n*_x_ = *M*). We sampled multi-dimensional output distributions in *M* = 1000 *d*-dimensional points obtained by running *M* simulations, each with parameters independently drawn from lognormal distributions as described in §2.1. The number of dimensions *d* was 2, 3, 4, 5, 6 or 8, equal to the number of pulses (or pseudopulses) of the considered sequences. The number of distinct binary sequences was 2*^d^*. From each sequence we obtained *d* numbers quantifying the ERK response to *d* subsequent pulses; see equation (3.1). We implemented the method of Kraskov *et al*. [19] such that for each input category, *x*, the counts of nearest neighbours from all other categories, *x′* ≠ *x*, are weighted according to current *p*(*x′*), enabling iterative constrained maximization of the estimated MI over the complete set of input probabilities. To obtain estimates of the maximum MI (channel capacity) and *p*(*x*) associated with inputs, we employed a stochastic gradient optimizer, Adam [20], available in TensorFlow [21]. Our implementation of the channel capacity estimator is provided in the form of a Python module called CCE (see http://pmbm.ippt.pan.pl/software/cce). The accuracy of our implementation of the Kraskov algorithm is checked within unit tests, where we calculate MI numerically based on eight three-dimensional Gaussian distributions either (1) centred along an S-shaped curve or (2) centred in vertices of the cube. In both cases the distributions are overlapping so MI is about 1.8 < log_2_(8) = 3 bit. These nearly accurate values can be then compared with the values obtained using the Kraskov algorithm based on *M* points drawn at random from each distribution. The Kraskov algorithm overestimates MI by about 2% for *M* = 1000 and 1.5% for *M* = 5000; in the second case, 3.5% for *M* = 1000 and 2.5% for *M* = 5000. Accuracy is nearly the same in the case when all eight input probabilities are equal (test_use_case_1.py) and in the case when input probabilities are varied to maximize MI (test_use_case_3.py).

## 3. Results

### 3.1. ERK responses to the repeated EGF pulses of different frequencies and amplitudes

The ability of a cell to respond to EGF pulses depends on their amplitude, duration and pulsing frequency. We focus on relatively short pulses of 5 min. In figure 1*b* we show trajectories resulting from one deterministic (black bold line) and 10 stochastic (thin blue lines) simulations of the MAPK model in response to two EGF pulses, both of amplitude 100 pg ml^−1^, separated by three different time intervals *T* of 45, 51.5 and 60 min. Let *R_i_*(*T*) denote the integrated ERK response after the *i*th EGF pulse:

3.1 $$\begin{array}{c} R_{i}(T)=\int_{iT}^{(i+1)T}ERK_{\;pp}(t)dt. \end{array}$$

Since the ERK_pp_ pulse lasts about 30 min, for *T* > 30 min, *R*_1_(*T*) = const = : *R*_max_ with the numerically calculated *R*_max_ = 2.65 × 10^9^ molecule × s. Let us consider the integrated ERK responses to the first and the second EGF pulse. For *T* = 45 min, the response to the second pulse, *R*_2_, is negligible with respect to *R*_1_, *R*_2_(45)/*R*_1_(45) ≪ 1, while for *T* = 51.5 min, *R*_2_(51.5)/*R*_1_(51.5) = 0.5. For *T* = 60 min, *R*_2_ = 2.00 × 10^9^ molecule × s is comparable to *R*_1_. This indicates a threshold *τ* = 51.5 min above (below) which the system can (cannot) be activated in response to the next pulse. It is of note that this threshold time (relaxation time) *τ* is very close to the minimal period of oscillations under constant EGF stimulation observed experimentally [3].

In figure 1*c*, we analyse the ERK activity time profile in response to EGF pulses repeated every 60 min, with successive amplitudes 3, 100, 10, 30 pg ml^−1^. While there is no response to the 3 pg ml^−1^ pulse, the responses to the remaining three pulses have comparable amplitudes, even though the 10 pg ml^−1^ pulse follows the much stronger 100 pg ml^−1^ pulse, which has an inhibitory effect. Overall, the results presented in figure 1*b*,*c* show that the MAPK system exhibits nearly all-or-nothing ERK responses to the pulsatile simulation. We thus expect that, for such stimulation, information is not coded by the level of EGF, but by the presence or absence of an EGF pulse that exceeds some threshold. Consequently, in the further analysis we restrict ourselves to 5 min long EGF pulses of amplitude 100 pg ml^−1^.

We notice that the stochastic trajectories in figure 1*b*,*c* are nearly indistinguishable from the deterministic one except for trajectories with *T* = 51.5 min in figure 1*b*. This is a consequence of a large number of reacting molecules at each step of the pathway, which makes the intrinsic noise of signal processing negligible. When comparing model predictions with experiment we found that, to reproduce experimentally observed heterogeneity of single cell responses, one has to include extrinsic noise, that is, assume that the levels of the MAPK pathway components vary among cells [4]. Since intrinsic noise was found to be negligible, henceforth we will use a deterministic approximation (ODEs). In order to analyse how extrinsic noise influences the transmitted information, we will consider two noise sources, as follows.

(a) Cell-specific noise associated with uncertain levels of pathway components (in individual simulated cells) and with pseudo-first-order rates of dephosphorylation reactions (by implicit phosphatases). We will assume that these variables follow the lognormal distributions logN(*μ_i_*, *σ*^2^) with median *μ_i_* equal to the default value of an *i*th parameter.
(b) Additive noise associated with ERK activation mediated by pathways that are not included in the model. For the additive noise we will also assume a lognormal distribution logN(μ*, $\sigma_{0}^{2}$) with *σ*_0_ = 1 and μ* = *μ*_0_ × *R*_max_ × *T*/(60 min). For most of the analysis we take *μ*_0_ = 0.03, which is equivalent to the assumption that about 3% of ERK activity results from the additive noise.

The parameters *σ* and *μ*_0_ that characterize the strength of the cell-specific and the additive noise will be varied. The MAPK channel information capacity (or simply bitrate) is controlled by the maximal frequency of EGF pulses that can induce ERK activation. For figure 1*d* we perform similar simulations to those for figure 1*b*, but accounting for the extrinsic noise. We compute the fraction of cells for which the response to the second pulse is at least half of the response to the first pulse, as a function of the time interval *T* between EGF pulses, for several values of *σ* (keeping *μ*_0_ = 0.03). For *σ* = 0, all cells exhibit significant responses to the second pulse when *T* > *τ* and none of them exhibit such responses when *T* < *τ*. For *σ* = 0.1 and *σ* = 0.3 the significant responses to the second EGF pulse are reached for *T* substantially larger than *τ*; additionally, even for *T* < *τ*, a fraction of cells exhibit significant responses to the second EGF pulse. For larger noise, *σ* ≥ 0.5, regardless of *T*, there are cells that exhibit a significant response to the second pulse and cells that fail to respond. In summary, based on the above analysis, we may expect that, for zero or small noise, EGF pulses separated by intervals *T* > *τ* will lead to a significant ERK activation, whereas, for larger noise, EGF pulses will be missed in some cells even for *T* > *τ*, but some other cells will show responses for *T* < *τ*.

### 3.2. ERK activation in response to sequences of three EGF pulses

**Figure 2.**
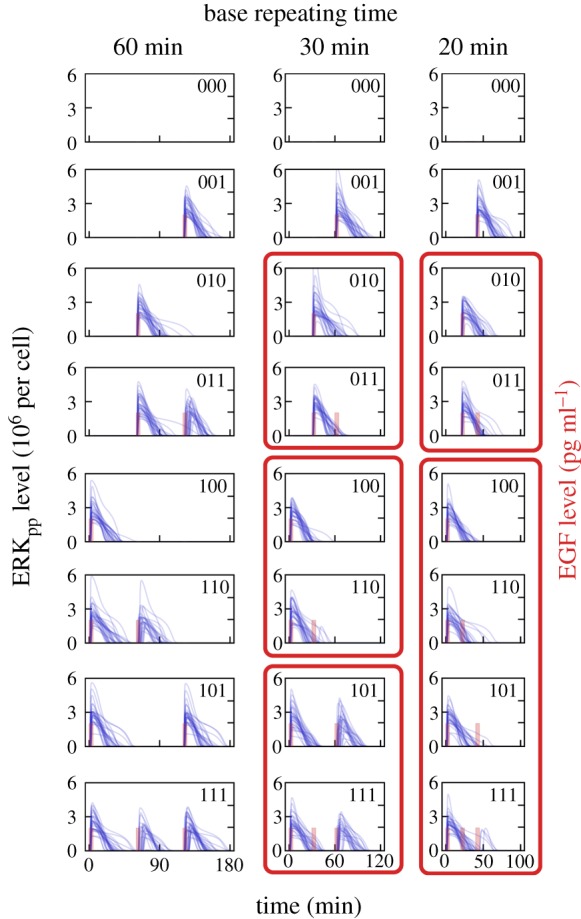
Responses to eight possible series of three pulses having amplitude of either ‘0’ (‘pseudo-pulse’) or ‘1’ (100 pg ml^−1^ in dimensional units). For each base repeating time *T_i_* (60, 30 or 20 min) and each pulse sequence, 30 independent numerical simulations were performed with *σ* = 0.3, *μ*_0_ = 0.03. For *T* = 30 min and *T* = 20 min, the sequences transmitted to the same output sequence are grouped and encircled by bold frames. The first element of each group is accurately transmitted and will be referred to as a group representative. (Online version in colour.)

In figure 2 we show responses to eight sequences of EGF pulses. We consider three base repeating times *T_i_* (time intervals between repeated pulses) of 60, 30 or 20 min. In the following convention the sequence ‘101’ for *T* = *T*_1_ = 60 min denotes a sequence of three EGF pulses of successive amplitudes: 1 (in dimensional units 1 × 100 pg ml^−1^) at *t* = 0 min, 0 (a ‘pseudo-pulse’) at *t* = 60 min, and 1 at *t* = 120 min. For *T* = *T*_2_ = 30 min, the same sequence denotes pulses of amplitude 1 at *t* = 0 min and *t* = 60 min, and a pseudo-pulse at *t* = 30 min.

For illustration purposes, times *T_i_* are chosen so that *T*_1_ > *τ*, *T*_2_ < *τ* < 2*T*_2_, and 2*T*_3_ < *τ* < 3*T*_3_. For each of the eight sequences and each *T_i_*, we perform 30 simulations (each corresponding to a single cell) with *σ* = 0.3, *μ*_0_ = 0.03; the resulting trajectories of *ERK_pp_*(*t*) are shown in figure 2. Since the noise is moderate, one can expect that for *T* = 60 min most cells will respond to all EGF pulses (by looking closely one can spot that only one and two cells show a very weak response to the second pulse in sequences ‘110’ and ‘111’, respectively). One can thus say that for *T* = 60 min almost all EGF sequences are transmitted properly.

For *T* = 30 min, cells are not able to properly transmit EGF sequences ‘011’, ‘110’ and ‘111’. Sequence ‘011’ leads only to a single ERK_pp_ pulse—in essence producing the same response as sequence ‘010’. We will say that these two input EGF sequences lead to a ‘010’ ERK_pp_ response. In some simplification, the distinction between ‘0’ and ‘1’ of the ERK_pp_ response is based on integrated ERK_pp_. When the integral exceeds some threshold, the ERK_pp_ response is interpreted as ‘1’. The threshold however is not predefined, but rather set by likelihood. Above the threshold the probability that the input was ‘1’ is higher than the probability that the input was ‘0’. EGF sequence ‘110’ is interpreted as ‘100’, whereas EGF sequence ‘111’ is interpreted as ‘101’. In the last case—because the second pulse does not elicit ERK activation—the third pulse can be transmitted. Overall, for *T* = 30 min, one can distinguish two EGF sequences, ‘000’ and ‘100’, that are accurately transmitted (i.e. produce ‘000’ and ‘100’ ERK response, respectively), and three groups {‘010’, ‘011’}, {‘100’, ‘110’} and {‘101’, ‘111’} (grouped in bold frames in figure 2) that are transcoded, respectively, to ERK_pp_ sequences ‘010’, ‘100’ and ‘101’. The element of the group which is accurately transmitted (i.e. here ‘010’, ‘100’ and ‘101’) will be referred to as the group representative. For *T* = 20 min, the analysis is analogous: there are also two sequences that are accurately transmitted, but only two groups—one containing two EGF sequences, the other containing four EGF sequences. Because an ERK_pp_ pulse at the first position inhibits signal transmission for about 50 min, EGF sequences ‘100’, ‘110’, ‘101’ and ‘111’ are all transcoded to ‘100’.

Transmitted information is limited from above by log_2_(*K*), where *K* is the number of distinct output sequences. In our case, *K* = 8 for *T* = 60 min, *K* = 5 for *T* = 30 min and *K* = 4 for *T* = 20 min. Thus, transmitted information (or MI) is limited from above by, respectively, log_2_(8) = 3, log_2_(5) = 2.32 and log_2_(4) = 2. The estimated bitrate, that is, MI/(3*T_i_*), equals, respectively, 1 bit h^−1^, 1.55 bit h^−1^ and 2 bit h^−1^. As we can see, the bitrate is highest when the base repeating time is 20 min, which is only a fraction of the relaxation time *τ*. We will see that this observation remains true also for longer sequences and that this simple theoretical estimates agree well with numerical values obtained for small noise.

### 3.3. Quantitative estimation of transmitted information

**Figure 3.**
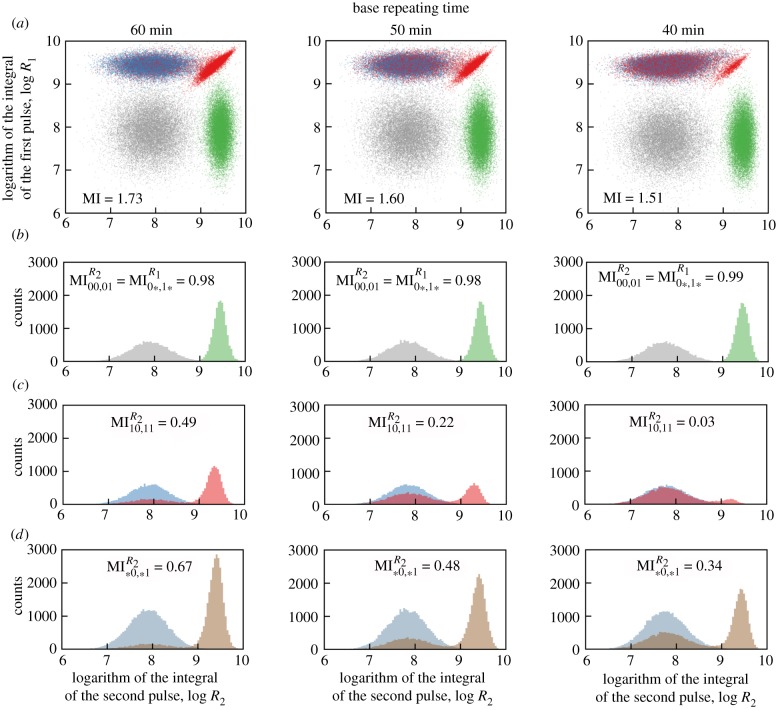
ERK responses to four binary sequences: 00, 01, 10 and 11 for three base repeating times. (*a*) Scatter plots of *R*_1_, *R*_2_ for all sequences (grey 00, green 01, blue 10, red 11), based on 16 000 simulations for each sequence. (*b*) Histograms of *R*_2_ when the first pulse has amplitude 0 (grey 00, green 01). (*c*) Histograms of *R*_2_ when the first pulse has amplitude 1 (blue 10, red 11). (*d*) Histograms of *R*_2_ independently of the first pulse amplitude (blue *0, brown *1). In all scatter plots, MI denotes the mutual information calculated for two-dimensional distributions; in all histograms, MI denotes the mutual information calculated based on two shown one-dimensional distributions.

In order to estimate the bitrate numerically we will calculate the MI between input and output sequences. The inputs x∈X are EGF binary sequences of length *L*. We have performed simulations for *L* = 8 and *L* = 6. For each input sequence we performed *M* = 1000 simulations obtaining *M* response vectors *y* = (*R*_1_, … ,*R*_L_) ∈ *Y*, where *R_i_* are defined as in equation (3.1) with the additive noise included. These simulations probe a (continuous) output probability distribution *Y*. The analysis of short sequences (*L* < 6) is based on truncated simulation data obtained for *L* = 6. In this way we gained larger statistics; for example, for *L* = 2 (figure 3), we have 16 000 simulations for each of four binary input sequences. In our case the domain of input is discrete, so MI can be expressed as

3.2 $$\begin{array}{c} \mathrm{MI}(X;Y)=\underset{x\in X}{\sum }p(x)\int_{y\in Y}\;p(y|x)\log\left(\frac{\;p(y|x)}{\;p(y)}\right)dy. \end{array}$$

To calculate MI, one has to assume or determine input sequence probabilities *p*(*x*). We proceed as follows:

(1) assume that all input sequences have equal probabilities, or
(2) assume that all group representatives have equal probabilities, while the other sequences have zero probabilities, or
(3) calculate input probabilities that maximize MI.

### 3.4. Numerical estimation of the transmitted information in sequences of two pulses

In figure 3 we analyse the ability of the system to resolve binary sequences ‘00’, ‘01’, ‘10’ and ‘11’ for three base repeating times *T_i_* = 60, 50 and 40 min, for moderate noise (*σ* = 0.3, *μ*_0_ = 0.03). In figure 3*a* we present scatter plots in the (*R*_1_, *R*_2_)-plane showing responses to the four binary sequences with corresponding MI values. The scatter plot for *T* = 40 min shows that for this time interval the system is essentially able to transmit three input sequences, ‘00’, ‘01’ and ‘10’, while the sequence ‘11’ produces almost the same output as sequence ‘10’. Consequently, MI = 1.51 < log_2_(3) ≈ 1.58. For *T* = 50 min and *T* = 60 min, the system distinguishes ‘10’ and ‘11’ sequences to a certain extent and MI values (equal, respectively, to 1.60 and 1.73) exceed log_2_(3) but are still substantially lower than the upper bound MI_max_ = log_2_(4) = 2.

We further investigate the system's inability to transmit four sequences by detailed analysis of responses to the second pulse. When the amplitude of the first pulse is 0, then, regardless of *T_i_*, the system almost perfectly distinguishes between amplitudes 0 and 1 of the second pulse (figure 3*b*). The value ${\mathrm{MI}}_{00,01}^{R_{2}}$ (given on each histogram in figure 3*b*) is calculated between the inputs *X* = {‘00’, ‘01’} (assuming that both sequences are equiprobable) and the output to the second pulse, *R*_2_. For each *T_i_*, ${\mathrm{MI}}_{00,01}^{R_{2}}$ ≥ 0.98 (${\mathrm{MI}}_{00,01}^{R_{2}}=1$ means that both inputs are perfectly distinguishable). In the case when the amplitude of the first pulse is 1, the ability to distinguish between amplitudes 0 and 1 of the second pulse is much lower (figure 3c): for *T* = 60 min, ${\mathrm{MI}}_{10,11}^{R_{2}}=0.49$; for *T* = 50 min, ${\mathrm{MI}}_{10,11}^{R_{2}}=0.22$; and for *T* = 40 min, ${\mathrm{MI}}_{10,11}^{R_{2}}=0.03$; the nearly zero value for *T* = 40 min is a consequence of the fact that the system cannot respond to two EGF pulses with a time interval of 40 min. In figure 3*d* we show histograms of the marginal probability distributions of responses to the second pulse. The ${\mathrm{MI}}_{*0,*1}^{R_{2}}$values shown in these histograms measure the ability to distinguish between amplitudes 0 and 1 of the second pulse when the first pulse is unknown.

Let us notice that two-dimensional MI is greater than the sum of unidimensional MIs, ${\mathrm{MI}}_{0*,1*}^{R_{1}}+{\mathrm{MI}}_{*0,*1}^{R_{2}}$. Here ${\mathrm{MI}}_{0*,1*}^{R_{1}}={\mathrm{MI}}_{00,01}^{R_{2}}$, because the response to the second pulse in the case when the first pulse has amplitude 0 is identical to the response to the first pulse—regardless of the amplitude of the second pulse. This implies that information that can be inferred from two pulses together is higher than information inferred from the first and the second pulse separately (i.e. when the interpretation of the second pulse ignores information inferred from the first pulse). This indicates that a receiver with memory, which can record and analyse the full response to an EGF sequence, can infer more information than a memoryless receiver that processes each pulse individually.

### 3.5. Theoretical versus numerical estimation of information carried by pulse sequences

To estimate MI for small noise, one can express it in terms of entropy and conditional entropy:

3.3 $$\begin{array}{c} \mathrm{MI}(Y;X)=H(Y)-H(Y|X)=H(X)-H(X|Y), \end{array}$$

where *H*(*X*) and *H*(*Y*) are the entropies of input and output, *H*(*Y*|*X*) is the conditional entropy of the output given the input, while *H*(*X*|*Y*) is the conditional entropy of the input given the output. First, we note that this formula implies that MI is limited by the input entropy *H*(*Y*) as well as the output entropy *H*(*X*). In the simplest case of *T* > *τ*, all EGF sequences are properly transmitted (transcoded) to ERK sequences, and thus *H*(*Y*|*X*) = *H*(*X*|*Y*) = 0 (which means that the knowledge of input is sufficient to deduce output and, conversely, knowledge of output is sufficient to infer input). Thus, for *T* > *τ* we have MI(*Y*; *X*) = *H*(*Y*) = *H*(*X*) = *L* (where, recall, *L* is the length of binary input sequences). In this case, the bitrate is equal to the base repeating frequency *Φ* = 1/*T*. In the case of *T* < *τ*, MI(*Y*; *X*) = *H*(*X*) can be reached when one is confined to input sequences that can be unambiguously transmitted, that is, to group representatives (as defined in figure 2).

Let us now consider the case of *L* = 4, *T* = 30 min. The input sequences form eight groups: {‘0000’}, {‘0001’}, {‘0010’, ‘0011’}, {‘0100’, ‘0110’}, {‘0101’, ‘0111’}, {‘1000’, ‘1100’}, {‘1001’, ‘1101’} and {‘1010’, ‘1110’, ‘1011’, ‘1111’}. In each group, input sequences are indistinguishable, so *K* = 8. The maximum MI is log_2_(8) = 3 and is achieved when probabilities of all group representatives (first sequence in each set) are equal to 1/8. The same maximal MI value is achieved when in each group the sum of all probabilities is equal to 1/8. In this case, one also learns 3 bits about input as knowing the output sequence allows one to infer from which of the eight equiprobable groups the input signal originates. Unsurprisingly, by maximizing MI numerically in the case of small noise (*σ* = 0.1, *μ*_0_ = 0.03) we obtain almost equal probabilities of each group of input sequences (table 1, last column).

**Table 1.** Information transmitted in *L* = 4 pulses with intervals *T* = 60, 30, 20, 15 min. Comparison of numerical and theoretical estimates for small noise, *σ* = 0.1 and *μ*_0_ = 0.03.

*T* (min)	theoretical MI value		numerical MI value, *σ* = 0.1 and *μ*_0_ = 0.03			maximum and minimum values of group probabilities together with the group representative^a^
	equal probabilities of input sequences	maximized log_2_(*K*)	numerically maximized	equal probabilities of all group representatives	equal probabilities of all input sequences	
60	log_2_(16) = 4	log_2_(16) = 4	3.93	3.93	3.93	‘0001’: 0.065; ‘0110’: 0.060
30	4–9/8 = 2.875	log_2_(8) = 3	2.96	2.96	2.82	‘0010’: 0.129; ‘1010’: 0.120
20	4–13/8 = 2.375	log_2_(6) = 2.58	2.55	2.55	2.33	‘0100’: 0.171; ‘1001’: 0.161
15	4–17/8 = 1.875	log_2_(5) = 2.32	2.32	2.32	1.89	‘1000’: 0.202; ‘0000’: 0.195

^a^Data obtained via numerical MI maximization.

Let us notice that by assuming that all 16 input sequences have equal probabilities, we obtain a somewhat lower value of MI(*Y*; *X*) = *H*(*X*) − *H*(*X*|*Y*). Now, *H*(*X*) = log_2_(16) = 4, while *H*(*X*|*Y*) = (2/16)log_2_(1) + (5/8)log_2_(2) + (1/4)log_2_(4) = 9/8, which gives MI(*Y*, *X*) = 23/8 < 3. The calculations for *T* = 20 min and *T* = 15 min are analogous, with the difference that for *T* = 20 min there are *K* = 6 groups, and for *T* = 15 min there are *K* = 5 groups. Also, in the two latter cases the maximized MI = log_2_(*K*) is larger than MI calculated under the assumption that all input sequences are equiprobable; moreover, the difference is larger than for *T* = 30 min. These theoretical values are compared with numerical estimates for small noise (table 1). One can see that both the maximized MI and the MI calculated by assuming that all sequences are equiprobable agree well with theoretical estimates. Additionally, one may notice that the numerically maximized MI perfectly agrees with the value calculated by assuming that all group representatives have equal probabilities and the other sequences have zero probability.

### 3.6. Bitrate estimation in the limit of infinitely long sequences

To estimate the upper limit of MI for small noise for a given base repeating time *T*, one has to calculate the number of sequences of length *L* that can be accurately transmitted. For *T* = 30 min, the accurately transmitted sequences are those without ‘11’ subsequences. It is straightforward to show that the numbers *n_L_* of such sequences follow the Fibonacci sequence. Let $n_{L}^{0}$ and $n_{L}^{1}$ denote the number of such sequences of length *L* ending in, respectively, ‘0’ and ‘1’. In the first case the sequence can be extended by adding either ‘0’ or ‘1’ at its end; in the second case it can be extended only by adding ‘0’. Thus, we have $n_{L+1}^{0}=n_{L}^{0}+n_{L}^{1}$, and $n_{L+1}^{1}\;=\;n_{L}^{0}$. Next, $n_{L+2}^{0}=n_{L+1}^{0}+n_{L+1}^{1}=n_{L+1}$ and $n_{L+2}^{1}=n_{L}^{0}+n_{L}^{1}=n_{L}$, hence *n_L_*_+2_ = *n_L_*_+1_ + *n_L_* (i.e. *n_L_*_+1_ = *n_L_* + *n_L_*_−1_). Analogously for *T* = 20 min (or any *T* satisfying 2 *T* < *τ* < 3 *T*), we may notice that the accurately transmitted sequences are those without both ‘11’ and ‘101’ subsequences. It is also straightforward to show that the number of such sequences satisfies a recurrence relation similar to the Fibonacci sequence, i.e. *n_L_*_+1_ = *n_L_* + *n_L_*_−2_. Finally, for any *T* satisfying *kT* < *τ* < (*k* + 1)*T*, the number of accurately transmitted sequences satisfies *n_L_*_+1_ = *n_L_* + *n_L_*_−_*_k_*. In the limit of large *L*, these recurrence relations can be solved: $n_{L}(k)\;=\;a_{k,0}\;\times \;a_{k}^{L}$, where *a_k_* satisfies $a_{k}^{k+1}=\;a_{k}^{k}+1$, and, for *k* ≤ 10, *a_k_*_,0_ are constants of order 1, i.e. *a*_1,0_ ≈ 1.17, *a*_2,0_ ≈ 1.03, *a*_3,0_ ≈ 0.94, *a*_10,0_ ≈ 0.74.

MI that can be transmitted in a sequence of length *L* is thus MI(*k*, *L*) = log_2_(*a_k_*_,0_) + *L* × log_2_(*a_k_*), therefore the bitrate *C*(*k*, *L*) = (*k* + 1)×MI(*k*, *L*)/*L* and, in the limit of *L* → ∞, *C*(*k*, *L*) = *C*(*k*) = (*k* + 1) × log_2_(*a_k_*). By straightforward calculation one can find that for *T* = 30 min the bitrate is *C*(2) ≈ 1.39 bit h^−1^; for *T* = 20 min, C(3) ≈ 1.65; and for *T* = 15 min, C(4) ≈ 1.86 bit h^−1^. *C*(*k*) is the monotonically increasing function of *k*, and *C*(*k*)/log_2_(*k*) → 1 for *k* → ∞.

**Figure 4.**
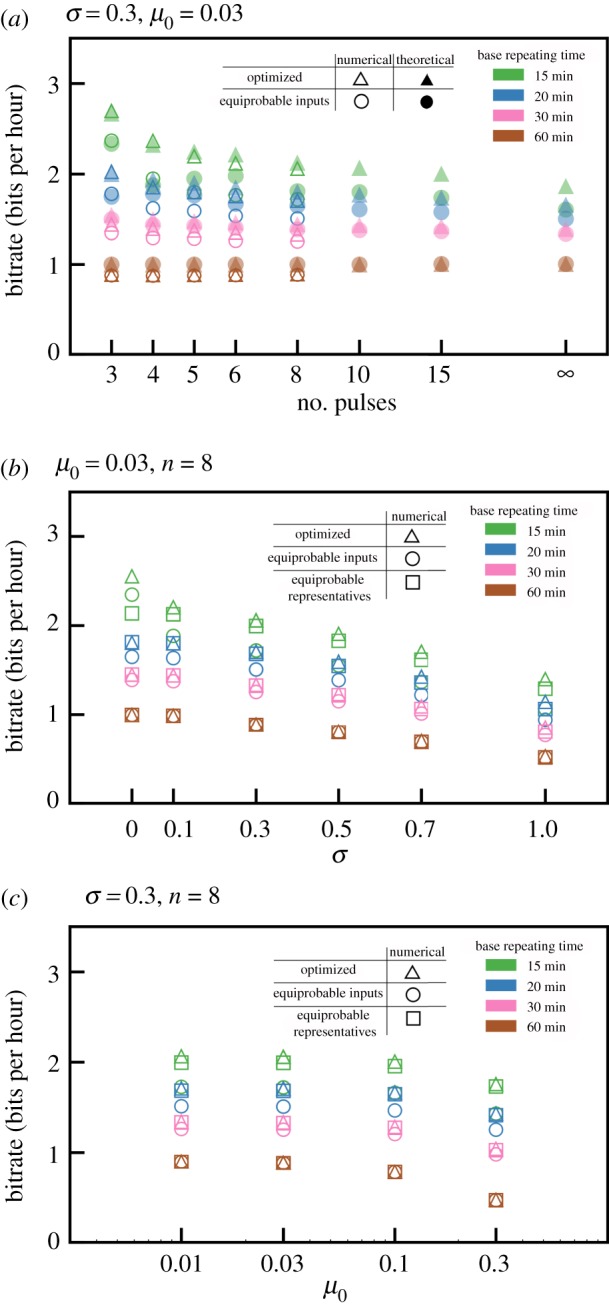
(*a*) Information transmitted per hour for moderate noise for different carrying frequencies *ϕ* = 1/*T*, where *T* = 15, 20, 30, 60 min. MI was calculated as follows: empty triangles—numerically optimized; solid triangles—optimized, i.e. log_2_(*K*); empty circles—numerically assuming equal probabilities of all input sequences; solid circles—theoretically assuming equal probabilities of all input sequences. (*b*) Influence of noise on transmitted information. (*c*) Influence of additive noise on transmitted information. In (*b*) and (*c*) MI was calculated numerically as follows: triangles—maximized; circles—assuming equal probabilities of all group representatives; squares—assuming equal probabilities of all input sequences.

In figure 4*a*, for inter-pulse intervals *T* = 60, 30, 20 and 15 min we compare bitrates that were predicted theoretically with those calculated based on numerical simulations. Despite a moderate magnitude of noise (*σ* = 0.3, *μ*_0_ = 0.03), there is good agreement between theoretical low-noise prediction and numerical simulations both for not maximized MI (equal input probabilities) and maximized MI. This suggests that the theoretical asymptotic bitrate value, *C*(*k*), serves as a good approximation even for low and moderate noise, *σ* ≤ 0.3 and *μ*_0_ ≤ 0.03.

Bitrates increase monotonically with repeating frequency *Φ* = 1/*T*. The maximized bitrate is higher than the bitrate calculated for equal input sequence probabilities. The difference is most significant for the smallest *T* = 15 min, when the ratio of sequences that can be properly transmitted to all sequences is low. However, even for equal input probabilities the bitrate for *T* = 15 min is much larger than for *T* = 60 min.

### 3.7. Influence of noise on bitrate

Noise can substantially decrease the amount of transmitted information. As shown in figure 1*d*, noise reduces a system's ability to respond to the second EGF pulse, especially when the time span between the pulses is comparable to *τ*. In figure 4*b* we show bitrate with respect to *σ*, with additive noise kept constant, *μ*_0_ = 0.03. As expected, bitrates for all considered *T* (60, 30, 20 and 15 min) decrease with *σ*. Interestingly, the ratio of the optimized bitrate for *T* = 15 min to the bitrate for *T* = 60 min increases with noise, which implies that coding using short base repeating time is less sensitive to noise. This is because, in the optimized coding using the short repeating times, the two (non-pseudo) pulses are less frequently separated by a 60 min interval than for the base repeating time *T* = 60 min. For *T* = 60 min, subsequences ‘10’ and ‘11’ are equiprobable, and the latter is sensitive to noise (figure 1*d*). Let us notice that even for very high noise parameter *σ* = 1.0, for *T* = 15 min, *C* > 1 bit h^−1^ (i.e. *C* exceeds the bitrate for *T* = 60 min in the limit of small noise).

Small noise *σ* = 0.1 influences bitrate only for *T* = 15 min but more detailed analysis suggests that for *T* = 15 min the high bitrate for *σ* = 0.0 can be considered an artefact. We found that in this case the system can distinguish between sequences ‘10’ and ‘11’, because the second EGF pulse increases the ERK activity tail. This effect is small and becomes invisible even for small noise (*σ* ≥ 0.1). The analysis shown in figure 4*b* is continued in electronic supplementary material, figure S1, where we consider the base repeating times from 30 to 80 min. For *σ* ≥ 0.3, bitrate increases monotonically with *Φ*; only for small noise, *σ* ≤ 0.1, is the bitrate for *T* = 60 min somewhat higher than for *T* = 50 min. This effect is clear in the context of figure 1*d*, which shows that for small noise the pathway transmits pulses separated by 60 min intervals but not by 50 min intervals.

In figure 4*c* we repeat the analysis shown in figure 4*b*, but now analysing the effect of the additive noise, keeping *σ* = 0.3. This effect is very weak up to *μ*_0_ = 0.1 (recall that for *μ*_0_ = 0.1 the amplitude of the additive noise is equal to 10% of the response amplitude). For *μ*_0_ = 0.3, the relative reduction of bitrate is significant, and the relative reduction again is the strongest for *T* = 60 min. The analysis is continued in electronic supplementary material, figure S2 for the base repeating times from 30 to 80 min and shows that, regardless of the amplitude of additive noise, bitrate increases monotonically with *Φ*.

In summary, the analysis shown in figure 4*b*,c and electronic supplementary material, figures S1 and S2 shows that, as expected, noise reduces bitrate. Generically, bitrate increases monotonically with *Φ* = 1/*T*, for *T* between 15 min and 80 min. Moreover, this increase is steeper for higher noise. In other words, high-frequency coding is less sensitive to noise than low-frequency coding.

## 4. Discussion

Two important regulatory pathways of NF-κB and ERK were found to transmit merely 1 bit of information about the level of extracellular stimulation [14,16], which roughly means that these pathways only relay information about the absence or presence of a stimulus. Information about the stimulation dose is increased only slightly when output is measured in more than one time point [16,22]. These results suggest that, in the considered pathways, information is encoded not in the input amplitude but in the temporal profile of, respectively, TNF and EGF, which are transcoded to the temporal profiles of nuclear NF-κB or active ERK. The question about optimal information coding can be formulated as the following representation problem: What is the input signal coding that allows the highest information transmission rate to be achieved in long time? It has been investigated previously whether the amplitude or the frequency coding can transduce more information from receptors to transcription factors [23]. Here, we proposed considering information coded in sequences of EGF pulses and, for such coding, we theoretically estimated from below the maximum transmission rate.

The considered model predicts that nearly all EGF inputs are converted to pulses of ERK activity [4]. Relaxation oscillations arise in response to constant EGF stimulation. The oscillation period is a non-monotonic function of the EGF level, attaining minima for the moderate stimulation. The ratio of the ON phase to the OFF phases increases monotonically with the EGF dose; at high dose, oscillations are replaced by constant response [4]. Analogous pulses of EGF are converted to nearly digital pulses of ERK activity. Conversion of various inputs to nearly digital ERK activity pulses may suggest that pulses are used as symbols in intracellular communication. This motivated us to quantify MI between sequences of short EGF pulses and corresponding sequences of ERK activity. Based on this we estimated from below the EGF-to-ERK-channel information capacity, or simply the bit rate at which information can be transmitted.

We showed that high repeating frequency coding allows to substantially exceed bandwidth limit equal 1 bit/*τ*, where, recall, *τ* ≈ 1 h is the threshold (or relaxation) time. Specifically, we considered sequences of EGF pulses of ‘1’ or ‘0’ amplitude separated by time intervals *T* that are multiples of hour/*n*, and showed that, in the case when intervals between pulses of amplitude ‘1’ (true pulses) are not smaller than 1 h, *C* ≈ 1.39 bit h^−1^ for *n* = 2, and *C* ≈ 1.86 bit h^−1^ for *n* = 4. Because ERK activity pulses are nearly digital, the considered encoding is not very sensitive to noise. Moreover, the high repeating frequency coding (*n* > 1) is less sensitive to noise than coding with *n* = 1. The high repeating frequency coding is equivalent to coding by pulse/spike intervals, proposed many years ago for neural information processing [24]. Neuronal spikes are separated by intervals of at least an order of magnitude longer than spike duration [25]. In this case, information is more efficiently encoded by time intervals between subsequent ‘1’ (that are not shorter than *τ* and resolved with accuracy *τ*/*n*) than by sequences of ‘0’ and ‘1’ separated by *τ*.

**Figure 5.**
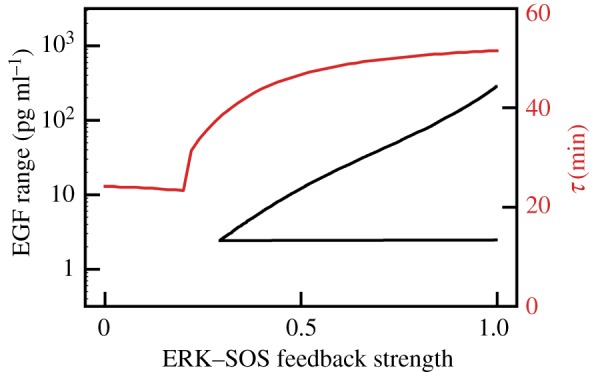
The range of EGF for which *ERK*_pp_ exhibits oscillations in response to constant EGF stimulation (black, lower line) and influence of the ERK-to-SOS feedback strength on threshold time *τ* (red, upper line). (Online version in colour.)

The bitrate is inversely proportional to threshold time, which for our model is *τ* = 51.5 min. It should be noted that the value of *τ* may be cell line dependent. According to the model, *τ* decreases with the strength of the negative feedback from ERK to SOS (figure 5). The abrupt decrease of the *τ* from 40 min to 25 min is observed when the strength of the feedback is close to one-quarter of its nominal value. At this value, ERK_pp_ does not exhibit oscillations in response to the constant EGF stimulation (figure 5). Therefore, the model suggests that there is a trade-off between short relaxation time and the ability of the system to translate constant EGF stimulation into series of pulses. In rat PC-12 cells, Ryu *et al*. [9] observed pronounced oscillations in response to 3 min long pulses with a period of 23 min, and also smaller oscillations (that are not arising in our model) in response to the higher frequency pulses. As expected from our analysis, these cells do not exhibit oscillations in response to a constant EGF stimulation. Responses exhibited by the PC-12 cell line can be reproduced by the considered model by removing the negative feedback from ERK to SOS and the positive feedback between SOS and RAS. This may suggest that signalling at the level of SOS is different in the PC-12 cell line from that in the human MCF-10A epithelial cells studied by us [4].

Because the negative feedback loop emanating from ERK targets SOS, a protein at the very beginning of the MAPK cascade, ERK activity oscillations are associated with oscillations of all components between SOS and MEK. Thus, the same or a higher amount of information is reached by pathway components preceding ERK. These proteins control different cellular functions. For example RAF-1 interacts with ROKα, which is implicated in the reorganization of cytoskeletal filaments [26] and with MST2/LATS pathway controlling apoptosis [27].

Characteristic spatio-temporal profiles of cytokines such as TNF and EGF have not been fully characterized. There is however growing evidence that these profiles are bursty and spatially localized rather than slowly fluctuating and spatially uniform. In the context of NF-κB, recently Bagnall *et al*. [28] showed that TNF propagation is of short range and NF-κB signalling is limited to distances of a few cell diameters from the neighbouring tissue-resident macrophages. Capuani *et al*. [29] demonstrated that at physiological EGF concentrations (which do not exceed 1 ng ml^−1^) only a fraction of EGF receptors are activated, which suggests that EGF diffusion is of short range, with EGF being removed from the extracellular space by the receptors. This allows for the existence of spatially localized ERK activity waves that may arise spontaneously [30] or in response to wounding [31], and coordinate motility of cells in the tissue. Hiratsuka *et al.* [31] observed *in vivo* ERK activation waves propagating from the wound edge. Under steady-state conditions, the epidermis occasionally revealed bursts of ERK activation patterns where ERK activation is propagated from cell to cell in a radial manner. ERK activity waves induce collective cell migration in the opposite direction [32], promoting wound healing or sealing a hole in the epithelium after individual cell apoptosis. These waves, although not yet fully characterized, are dependent on EGF receptor ligands because inhibition of EGF receptors completely blocks ERK activity propagation [27], which in turn suppresses collective cell migration [32]. Propagation of ERK activity waves requires timely digital ERK activation in subsequent layers (or lines in epithelium) of cells. To maintain directionality cells behind the wavefront have to remain inhibited until the signal propagates to the next layer. The ability to transmit spontaneous signals requires a single cell to accurately respond to random sequences of EGF pulses, which is equivalent to transmitting information at sufficiently high bit rates.

Regulatory pathways can be perceived as noisy information-transmitting channels, but can also be considered as decision-making modules that employ feedbacks and other nonlinear regulatory elements to convert input into one of several predefined outputs. MAPK/ERK and NF-κB pathways convert both constant and pulsating cytokine stimulation into pulses of ERK or NF-κB activity. Here, we consider an optimal representation problem and investigate which EGF time profiles can transmit the maximum amount of information to ERK. Motivated by the growing evidence that physiological EGF stimulation is short ranged and bursty, we focus on sequences of short EGF pulses. We found that information can be transmitted with the rate exceeding the classical bandwidth limit of about 1 bit h^−1^ in the case when inter-pulse intervals are used to code information. The timely activation of ERK in neighbouring cells allows for propagation of waves that coordinate collective cell migration during wound healing.

## Supplementary Material

Supplementary Figures and Table

## Data accessibility

All data supporting the presented results are included in the article or electronic supplementary material. The source code of our Python module for estimation of information channel capacity is available at http://pmbm.ippt.pan.pl/software/cce.

## References

[1] Kellogg RA, Tian C, Lipniacki T, Quake SR, Tay S 2015 Digital signaling decouples activation probability and population heterogeneity. Elife 4, e08931 (10.7554/eLife.08931) 26488364 PMC4608393

[2] Hao N, O'Shea EK 2011 Signal-dependent dynamics of transcription factor translocation controls gene expression. Nat. Struct. Mol. Biol. 19, 31–39. (10.1038/nsmb.2192) 22179789 PMC3936303

[3] Albeck JG, Mills GB, Brugge JS 2013 Frequency-modulated pulses of ERK activity transmit quantitative proliferation signals. Mol. Cell 49, 249–261. (10.1016/j.molcel.2012.11.002) 23219535 PMC4151532

[4] Kochańczyk M, Kocieniewski P, Kozłowska E, Jaruszewicz-Błońska J, Sparta B, Pargett M, Albeck JG, Hlavacek WS, Lipniacki T 2017 Relaxation oscillations and hierarchy of feedbacks in MAPK signaling. Sci. Rep. 7, 38244 (10.1038/srep38244) 28045041 PMC5206726

[5] Behar M, Hoffmann A 2010 Understanding the temporal codes of intra-cellular signals. Curr. Opin. Genet. Dev. 20, 684–693. (10.1016/j.gde.2010.09.007) 20956081 PMC2982931

[6] Levine JH, Lin Y, Elowitz MB 2013 Functional roles of pulsing in genetic circuits. Science 342, 1193–1200. (10.1126/science.1239999) 24311681 PMC4100686

[7] Purvis JE, Lahav G 2013 Encoding and decoding cellular information through signaling dynamics. Cell 152, 945–956. (10.1016/j.cell.2013.02.005) 23452846 PMC3707615

[8] Tay S, Hughey JJ, Lee TK, Lipniacki T, Quake SR, Covert MW 2010 Single-cell NF-κB dynamics reveal digital activation and analogue information processing. Nature 466, 267–271. (10.1038/nature09145) 20581820 PMC3105528

[9] Ryu H et al. 2015 Frequency modulation of ERK activation dynamics rewires cell fate. Mol. Syst. Biol. 11, 838 (10.15252/msb.20156458) 26613961 PMC4670727

[10] Ashall L et al 2009 Pulsatile stimulation determines timing and specificity of NF-κB-dependent transcription. Science 324, 242–246. (10.1126/science.1164860) 19359585 PMC2785900

[11] Korwek Z, Tudelska K, Nałęcz-Jawecki P, Czerkies M, Prus W, Markiewicz J, Kochańczyk M, Lipniacki T 2016 Importins promote high-frequency NF-κB oscillations increasing information channel capacity. Biol. Direct 11, 61 (10.1186/s13062-016-0164-z) 27835978 PMC5106790

[12] Shannon CE 1948 A mathematical theory of communication. Bell Syst. Tech. J. 27, 379–423. (10.1002/j.1538-7305.1948.tb01338.x)

[13] Shannon CE 1948 A mathematical theory of communication. Bell Syst. Tech. J. 27, 623–656. (10.1002/j.1538-7305.1948.tb00917.x)

[14] Cheong R, Rhee A, Wang CJ, Nemenman I, Levchenko A 2011 Information transduction capacity of noisy biochemical signaling networks. Science 334, 354–358. (10.1126/science.1204553) 21921160 PMC3895446

[15] Uda S, Saito TH, Kudo T, Kokaji T, Tsuchiya T, Kubota H, Komori Y, Ozaki Y, Kuroda S 2013 Robustness and compensation of information transmission of signaling pathways. Science 341, 558–561. (10.1126/science.1234511) 23908238

[16] Selimkhanov J, Taylor B, Yao J, Pilko A, Albeck J, Hoffmann A, Tsimring L, Wollman R 2014 Accurate information transmission through dynamic biochemical signaling networks. Science 346, 1370–1373. (10.1126/science.1254933) 25504722 PMC4813785

[17] Tudelska K et al 2017 Information processing in the NF-κB pathway. Sci. Rep. 7, 15926 (10.1038/s41598-017-16166-y) 29162874 PMC5698458

[18] Faeder JR, Blinov ML, Hlavacek WS 2009 Rule-based modeling of biochemical systems with BioNetGen. In Systems biology (ed. Maly IV), pp. 113–167. Totowa, NJ: Humana Press. 10.1007/978-1-59745-525-1_5 19399430

[19] Kraskov A, Stögbauer H, Grassberger P 2004 Estimating mutual information. Phys. Rev. E 69, 066138. 10.1103/PhysRevE.69.066138 15244698

[20] Kingma DP, Ba J 2014 Adam: a method for stochastic optimization. In Proceedings of the 3rd International Conference on Learning Representations (ICLR), *San Diego, CA, USA, May 7–9, 2015*.

[21] Abadi M et al 2015 *TensorFlow: large-scale machine learning on heterogeneous systems*. See https://www.tensorflow.org/.

[22] Potter GD, Byrd TA, Mugler A, Sun B 2017 Dynamic sampling and information encoding in biochemical networks. Biophys. J. 112, 795–804. (10.1016/j.bpj.2016.12.045) 28256238 PMC5340174

[23] Hansen AS, O'Shea EK 2015 Limits on information transduction through amplitude and frequency regulation of transcription factor activity. Elife 4, e06559 (10.7554/eLife.06559) PMC4468373 25985085

[24] MacKay DM, McCulloch WS 1952 The limiting information capacity of a neuronal link. Bull. Math. Biophys. 14, 127–135. (10.1007/BF02477711)

[25] Cessac B, Paugam-Moisy H, Vieville T 2010 Overview of facts and issues about neural coding by spikes. J. Physiol. Paris 104, 5–18. (10.1016/j.jphysparis.2009.11.002) 19925865

[26] Varga A, Ehrenreiter K, Aschenbrenner B, Kocieniewski P, Kochanczyk M, Lipniacki T, Baccarini M 2017 RAF1/BRAF dimerization integrates the signal from RAS to ERK and ROKα. Sci. Signal. 10, eaai8482 (10.1126/scisignal.aai8482) 28270557

[27] Romano D, Nguyen LK, Matallanas D, Halasz M, Doherty C, Kholodenko BN, Kolch W 2014 Protein interaction switches coordinate Raf-1 and MST2/Hippo signalling. Nat. Cell Biol. 16, 673–684. (10.1038/ncb2986) 24929361

[28] Bagnall J et al 2018 Quantitative analysis of competitive cytokine signaling predicts tissue thresholds for the propagation of macrophage activation. Sci. Signal. 11, eaaf3998 (10.1126/scisignal.aaf3998) 30042130

[29] Capuani F, Conte A, Argenzio E, Marchetti L, Priami C, Polo S, Di Fiore PP, Sigismund S, Ciliberto A. 2015 Quantitative analysis reveals how EGFR activation and downregulation are coupled in normal but not in cancer cells. Nat. Commun. 6, 7999 (10.1038/ncomms8999) 26264748 PMC4538861

[30] Aoki K, Kumagai Y, Sakurai A, Komatsu N, Fujita Y, Shionyu C, Matsuda M 2013 Stochastic ERK activation induced by noise and cell-to-cell propagation regulates cell density-dependent proliferation. Mol. Cell 52, 529–540. (10.1016/j.molcel.2013.09.015) 24140422

[31] Hiratsuka T, Fujita Y, Naoki H, Aoki K, Kamioka Y, Matsuda M 2015 Intercellular propagation of extracellular signal-regulated kinase activation revealed by *in vivo* imaging of mouse skin. Elife 4, e05178 (10.7554/eLife.05178) 25668746 PMC4337632

[32] Aoki K, Kondo Y, Naoki H, Hiratsuka T, Itoh RE, Matsuda M 2017 Propagating wave of ERK activation orients collective cell migration. Dev. Cell 43, 305–317.e5. (10.1016/j.devcel.2017.10.016) 29112851

