# Supplementary material for: Limits to the rate of information transmission through the MAPK pathway
Source: J R Soc Interface. 2019 Mar 6;16(152):20180792. doi: 10.1098/rsif.2018.0792 (PMC6451410; doi:10.1098/rsif.2018.0792)
Supplement: Supplementary Figures and Table [file rsif20180792supp1.pdf]

Supplementary materials

featuring article

Limits to the rate of information transmission through MAPK pathway

by

Frederic Grabowski[1], Paweł Czyż[2], Marek Kochańczyk[3] and Tomasz Lipniacki[3]

[1]Faculty of Mathematics, Informatics and Mechanics, University of Warsaw, Poland,

[2]University of Oxford, Oxford, UK,

[3]Institute of Fundamental Technological Research, Polish Academy of Sciences, Warsaw, Poland

Contents

Supplementary Figure 1. Influence of extrinsic noise on transmitted information.

Supplementary Figure 2. Influence of additive noise on transmitted information.

Supplementary Table 1. Parameter values for the MAPK pathway model.

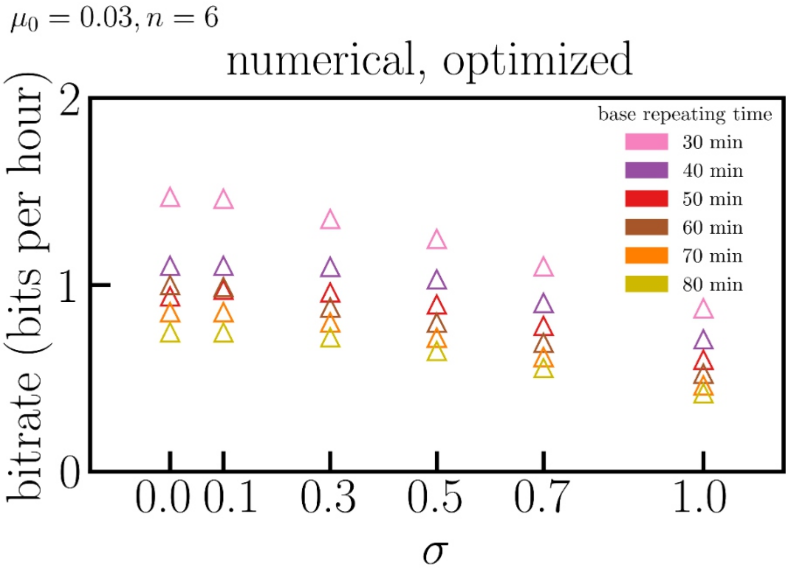

Supplementary Figure 1. Influence of cell specific noise on transmitted information. Mutual information was calculated numerically as follows: triangles – maximized, circles – assuming equal probabilities of all group representatives, squares – assuming equal probabilities of all input sequences.

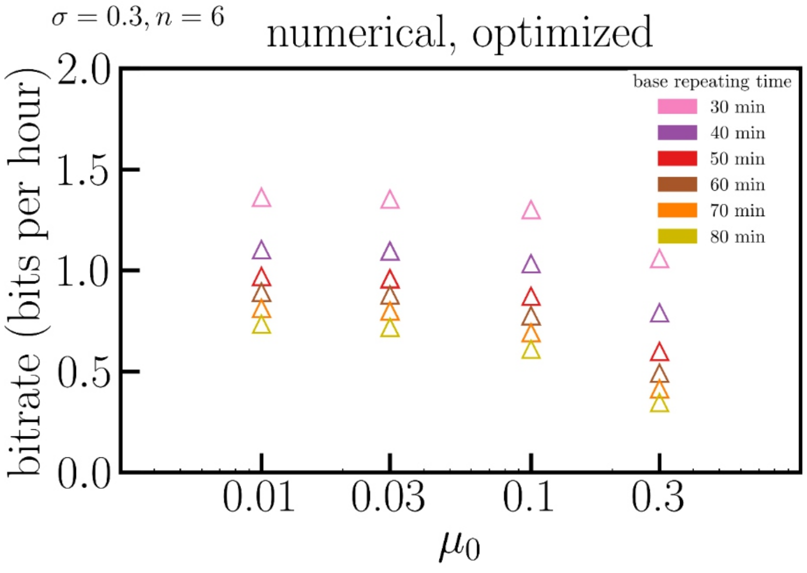

Supplementary Figure 2. Influence of additive noise on transmitted information. Mutual information was calculated numerically as follows: triangles – maximized, circles – assuming equal probabilities of all group representatives, squares – assuming equal probabilities of all input sequences.

Supplementary Table 1. Parameter values for the MAPK pathway model.

Default values originate from the article by Kochańczyk *et al.* (2017), *Sci. Rep.*, **7**:38244, doi:10.1038/srep38244. The parameters of the pathway that are drawn from the lognormal distribution LogN(μ_i, σ) are written in bold green.

Parameter	Description
Concentrations (protein copy number per cell)	
**$EGFR_{tot} = 3 \times 10^5$**	Total number of EGFR molecules
**$RAS_{tot} = 6 \times 10^4$**	Total number of RAS molecules
**$SOS_{tot} = 10^5$**	Total number of SOS molecules
**$RasGAP_{tot} = 6 \times 10^3$**	Total number of RasGAP molecules
**$RAF_{tot} = 5 \times 10^5$**	Total number of RAF molecules
**$MEK_{tot} = 2 \times 10^5$**	Total number of MEK molecules
**$ERK_{tot} = 3 \times 10^6$**	Total number of ERK molecules
Rate constant for fast processes (s^{-1})	
$k_{fast} = 100$	As a simplification, three (pseudo) first-order processes considered in the model are assumed to be fast.
Rate constants for activation processes [(molecules/cell)$^{-1}$ s^{-1} except (pg/ml)$^{-1}$ s^{-1} for a_1]	
$a_1 = 5 \times 10^{-5}$	EGF-mediated activation of EGFR
$a_2 = 10^{-7}$	Activation of RAF by RAS-GTP
$b_1 = 10^{-5}$	Binding of EGF-activated EGFR and dephosphorylated SOS
$p_1 = 10^{-7}$	Phosphorylation of MEK's activation sites by RAF
$p_2 = 3 \times 10^{-6}$	Phosphorylation of ERK's activation sites by MEK
Rate constants for deactivation processes (s^{-1})	
$d_1 = d = 0.01$	Deactivation of EGFR
**$d_2 = d$**	Deactivation of RAF (spontaneous)
$u_{1A} = d$	Dissociation of active EGFR–SOS complex
**$q_1 = d$**	Dephosphorylation of MEK's activation sites
**$q_2 = d$**	Dephosphorylation of ERK's activation sites
Rate constants for SOS association reactions [(molecules/cell)$^{-1}$ s^{-1}] *and dissociation reactions* (s^{-1})	

$b_{2A} = 10^{-6}$	Association of SOS and RAS-GTP
$b_{2B} = 0.1 \times b_{2A}$	Association of SOS and RAS-GDP
$u_{1B} = k_{fast}$	Dissociation of inactive EGFR–SOS complex
$u_{2A} = u_{2B} = 1$	Dissociation of SOS–RAS-GTP complex and SOS–RAS-GDP complex
Rate constants associated with SOS-to-RAS positive feedback [(molecules/cell)$^{-1}$ s^{-1} except s^{-1} for k_3 and u_3]	
$k_{2A} = 10^{-4}$	SOS's nucleotide-exchange activity when RAS-GTP is bound to its REM domain
$k_{2B} = 0.1 \times k_{2A}$	SOS's nucleotide-exchange activity when RAS-GDP is bound to its REM domain
$k_{2C} = 0$	SOS's nucleotide-exchange activity when no RAS is bound to its REM domain
$b_3 = 10^{-5}$	Association of RasGAP and RAS-GTP
$k_3 = k_{fast}$	RasGAP-facilitated RAS-GTP→RAS-GDP hydrolysis
$u_3 = 0.01$	Dissociation of RasGAP from RAS-GDP after hydrolysis of GTP
Rate constants associated with ERK-mediated negative feedbacks [(molecules/cell)$^{-1}$ s^{-1}]	
$p_3 = 3\times10^{-9}$	Phosphorylation of unbound SOS by active ERK
$p_4 = p_6 = p = 6\times10^{-10}$	Phosphorylation of MEK (T292 in MEK1) and RAF by active ERK
Rate constants for (spontaneous) dephosphorylation reactions (s^{-1})	
**$q_3 = q_4 = q_6 = q = 3\times10^{-4}$**	Dephosphorylation of inhibitory phosphosites in SOS, MEK (T292 in MEK1), RAF
**$q_5 = k_{fast}$**	Dephosphorylation of MEK's activation sites when MEK's T292 is phosphorylated
ERK activity reporters – concentrations (protein copy number per cell)	
$EKAR3_{tot} = ERKTR_{tot} = 10^6$	Total number of EKAR3 and ERKTR molecules
ERK activity reporters – rate constants for activation reactions [(molecules/cell)$^{-1}$ s^{-1}]	
$a_{EKAR3} = 3\times10^{-9}$	EKAR3 activation rate by active ERK
$a_{ERKTR} = 10^{-9}$	ERKTR activation rate by active ERK
ERK activity reporters – rate constants for deactivation reactions (s^{-1})	
$d_{EKAR3} = 10^{-3}$	EKAR3 deactivation rate
